# A balancing act of the brain: activations and deactivations driven by cognitive load

**DOI:** 10.1002/brb3.128

**Published:** 2013-04-02

**Authors:** Marie Arsalidou, Juan Pascual-Leone, Janice Johnson, Drew Morris, Margot J Taylor

**Affiliations:** 1 Diagnostic Imaging and Research Institute, Hospital for Sick Children, University of TorontoToronto, Ontario, Canada; 2 Department of Psychology, York UniversityToronto, Ontario, Canada

**Keywords:** Default mode, difficulty, fMRI, working memory

## Abstract

The majority of neuroimaging studies focus on brain activity during performance of cognitive tasks; however, some studies focus on brain areas that activate in the absence of a task. Despite the surge of research comparing these contrasted areas of brain function, their interrelation is not well understood. We systematically manipulated cognitive load in a working memory task to examine concurrently the relation between activity elicited by the task versus activity during control conditions. We presented adults with six levels of task demand, and compared those with three conditions without a task. Using whole-brain analysis, we found positive linear relations between cortical activity and task difficulty in areas including middle frontal gyrus and dorsal cingulate; negative linear relations were found in medial frontal gyrus and posterior cingulate. These findings demonstrated balancing of activation patterns between two mental processes, which were both modulated by task difficulty. Frontal areas followed a graded pattern more closely than other regions. These data also showed that working memory has limited capacity in adults: an upper bound of seven items and a lower bound of four items. Overall, working memory and default-mode processes, when studied concurrently, reveal mutually competing activation patterns.

## Introduction

Functional neuroimaging studies typically manipulate cognitive demand of tasks by changing executive load (e.g., n-back tasks; Owen et al. 2005 for meta-analysis) or number of items on the display over a temporal delay (e.g., Sternberg tasks; Manoach et al. 1997; Rypma et al. 1999, 2002; Jha and McCarthy 2000). Researchers have also identified a set of areas that are active when a cognitive task is not required, during rest (e.g., Spreng et al. 2009 for meta-analysis). Knowledge of the brain areas that underlie cognitive load versus rest activity is expanding, but their interrelation is not well understood. We used functional magnetic resonance imaging (fMRI) and a working memory task with graded increases in cognitive load (Arsalidou et al. 2010), to examine, using linear regression, whole-brain changes in activity as a function of task difficulty.

A classic working memory protocol used to manipulate cognitive load is the n-back task. In a typical n-back paradigm, participants view a series of stimuli and indicate whether the current stimulus matches the stimulus *n* items earlier in the series. As *n* increases, the number of interpolated stimuli between criterion and target increases, and thus cognitive load increases. Cognitive demand increases qualitatively (e.g., 0-back, recognition, 1-back, maintenance, 2-back, maintenance and monitoring), and because cognitive load increases nonlinearly from one level to the next, these changes are not easily quantifiable. In a coordinate-based meta-analysis of 24 n-back studies, Owen et al. (2005) identified six cortical regions that were reliably activated by n-back tasks. In prefrontal regions, activity was typically elicited in inferior frontal (BA 45/47), middle frontal (BA 9/46), and anterior medial frontal gyri (BA 10; Owen et al. 2005). Other areas included the dorsal cingulate gyrus (BA 32), the premotor cortex (BA 6), and parietal regions (BA 7/40; Owen et al. 2005). In this study, by manipulating cognitive load, we expected to replicate this set of areas typically found with adults, and also show how brain activity elicited by graded increases in cognitive load might also affect activity found in the control conditions (i.e., no task).

Shulman et al. (1997) were probably first to use fMRI to study brain activity associated with no task, usually referred to as control/rest condition. Subsequent work has confirmed the importance of brain activity not externally driven by problem-solving tasks – activity typically referred to as default mode of processing (Raichle et al. 2001; Greicius et al. 2003; Persson et al. 2007; Buckner et al. 2008). In a meta-analysis of 16 studies, Spreng et al. (2009) discussed brain responses associated with task-related deactivations, or activations associated with rest or fixation. The medial prefrontal cortex (BA 10, 11, 32), the temporal parietal junction (BA 39, 22), and the posterior cingulate (BA 31) adjacent to the medial precuneus (BA 7) showed the highest likelihood of being active during control/rest tasks or task deactivations.

Our understanding of the default-mode areas is evolving. For instance, research suggests that when working memory areas are more active, default-mode regions are less active (Persson et al. 2007). Similarly, negative correlations between the two processes were observed in a single-difficulty task that focused on intraindividual differences (Kelly et al. 2008). Although speculation and converging evidence may suggest that task-positive and task-negative activation have an inverse linear relation, there has not yet been a direct experimental observation of this effect. One way to examine this effect is by graded variation of the cognitive load, measuring brain activity concurrently in the working memory and default-mode systems. To date, only a few studies have used several levels of task difficulty to examine association between task difficulty and brain deactivations (McKiernan et al. 2003, 2006; Singh and Fawcett 2008). With only three memory loads (McKiernan et al. 2003, 2006) or a perceptual paradigm (Singh and Fawcett 2008), these studies reported decreases in brain activity, but did not explicitly report brain activity that increased in a graded manner with increases in task difficulty.

Task difficulty is better maintained and controlled in working memory tasks that contain irrelevant cues, which are features in a task that may interfere with performance (Pomerantz and Garner 1973). Huettel and Lockhead (1999) provided a comprehensive classification of tasks that are used to investigate variations of irrelevant perceptual dimensions (e.g., size, orientation). What is sometimes referred to as “Garner interference” (Pomerantz and Garner 1973; Garner 1974) suggests that it takes longer to classify a relevant item in the presence of variations of irrelevant dimensions, than in their absence. Further, behavioral work suggests that irrelevant cues in a task improve the assessment of working memory (Pascual-Leone and Baillargeon 1994; Engle 2001; Engle and Kane 2004; Arsalidou et al. 2010). Thus, in designing the current task, we introduced irrelevant features and found that with their inclusion, the task was better able to assess working memory, compared with a similar task with minimal interference (Arsalidou et al. 2010). Difficulty in the current task was manipulated by varying the number of relevant cues while keeping irrelevant cues constant across six levels of difficulty.

The current task can be contrasted with two popular measures of working memory (e.g., n-back; Owen et al. 2005) and the Sternberg tasks (Sternberg 1966; Manoach et al. 2003). Although a working memory task, our task differs in theoretically interesting ways from the classic paradigms that gave it an advantage for answering our hypotheses. We used a variant of a 1-back task in which difficulty in cognitive processes increased with the number of relevant cues, in this case colors. In terms of cognitive load and methodology, what sets our fMRI research paradigm apart is the following: First, difficulty was parametrically graded across classes of items (according to theoretical modeling and prior developmental work, Arsalidou et al. 2010). Second, executive demand was controlled (i.e., constant across levels). Third, most other imaging studies fail to consider a sufficient number of graded difficulty levels (Rypma et al. 2002 being an exception in the verbal domain). Without these many levels, it is impossible to account for the capacity limitations in mental attention proposed by both working memory (Cowan 2005) and developmental researchers (e.g., Pascual-Leone 1970; Halford et al. 1998). Fourth, in terms of statistical power, the current task was designed as a block paradigm with relatively short trials in order to accommodate six levels of difficulty. In this regard, it should be noted that fMRI studies that have many conditions face a trade-off between the number of trials needed for sufficient statistical power and the time participants can stay in the scanner, particularly so in studies with children (Gaillard et al. 2001).

The range of levels of working memory capacity that can be assessed using our tasks is very relevant for the study of developmental and clinical populations. With a future aim to use the tasks for neuroimaging with developmental populations, we were interested in methods that minimize extraneous developmental-laden factors (we used short runs, child friendly content, etc.; see Gaillard et al. 2001; Luna et al. 2010). To facilitate comparisons across populations, Luna et al. (2010) recommended the use of tasks with well-understood neural correlations in the adult literature. Thus, prior to this study, our working memory task was validated behaviorally in adults as well as in children (Arsalidou et al. 2010). Behavioral performance followed a graded age-dependent growth pattern such that 7–8, 9–10, 11–12, 13–14 year olds, and adults could cope with working memory demands up to 3, 4, 5, 6, and 7 units, respectively (Arsalidou et al. 2010). These observations point to a linear pattern in working memory development that is captured by our task. It is on this basis that our current hypotheses and analyses investigate particularly a linear pattern. If a linear pattern is present in performance across development, it may also be observed in the relation between neural processes of the working memory and default systems.

In this study, we use the working memory task designed by Arsalidou et al. (2010) to study the possible covariation between task difficulty and task-based cortical activations, as well as a possible concomitant deactivation found under control conditions (default mode) in adults. If working memory and default-mode activities are present within each difficulty level, we could investigate their association by varying task demand to examine how the brain activity elicited by working memory and default-mode processes are related. Specifically, we expected activity to be linearly modulated (directly for working memory areas, inversely in default-mode areas) by difficulty levels in the task. Thus, as behavioral performance improved linearly across development (Arsalidou et al. 2010), we expected to see a linear increase in activity related to mnemonic processes as a function of difficulty and also a concurrent linear decrease in activity in areas related to the default mode.

## Materials and Methods

### Participants

Data were collected from 10 right-handed adult volunteers (six females, *mean age* = 28.06 ± 3.8 years), recruited from research labs at the Hospital for Sick Children (Toronto, ON, Canada). Participants had 16 or more years of formal education. Exclusion criteria included color blindness – tested during pretraining – and ferromagnetic implants or history of neurological disorders. Procedures were approved by the research ethics boards at York University and the Hospital for Sick Children; all participants signed informed consent.

### Measures

#### Color matching task

**Figure 1:**
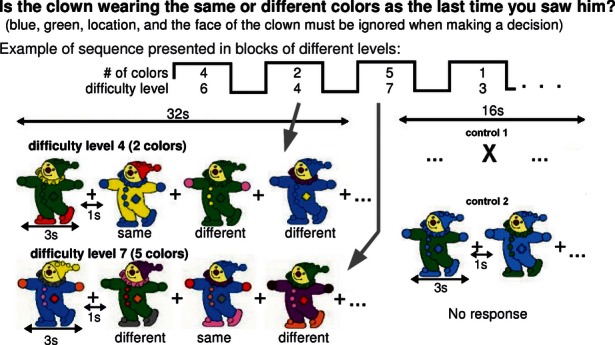
Example of sequence presentation and stimuli for color matching task (CMT)-clown. In a 1-back design, participants indicated in sequence whether or not the present clown had the same or different relevant colors as the previous clown. During training they learned that all colors were relevant except blue and green. Task blocks (32 sec) were interleaved by control blocks (16 sec). Difficulty, the item's mental demand, equals 2 plus the number of colors in the figure; the number of relevant colors ranged from 1 to 6; thus, difficulty ranged from D3 to D8 (Arsalidou et al. 2010 for more details). Blocks were ordered pseudo-randomly. No response was required for control items. Control 1 was a fixation cross; control 2 clowns were colored blue and green (i.e., irrelevant colors); control 3 (not shown) clowns were colored blue and green with a dot appearing in various locations (rate of 1 Hz).

The color matching task (CMT) was designed in two versions (Arsalidou et al. 2010). CMT-balloon was administered on a personal computer as training for the CMT-clown (Fig. 1), which was administered in the MR scanner. The template figure was, respectively, a set of balloons or a clown. Both figures had different parts colored (using 1–6 colors; yellow, purple, pink, orange, brown, red, and gray; with the added base colors blue and green, both irrelevant and to be ignored for the task). The number of relevant colors in each figure indexed item difficulty. Color location was not relevant and changed between successive figures. The clown's faces also had to be ignored as irrelevant. Participants were asked to indicate whether the current figure contained the same relevant colors as the previous figure. Task difficulty equalled *n* + 2 for CMT-clown and *n* + 1 for CMT-balloon, where *n* corresponded to the number of relevant colors. The additional cognitive demand was based on executive schemes: (a) in both tasks participants also have to remember the goal of the task (+1) and (b) in the CMT-clown participants needed to extract relevant cues while ignoring features like the face and different shapes on the outfit (+1). For detailed task analyses see Arsalidou et al. (2010). There was a 50% chance of an item containing the same colors as the previous item (i.e., half of the correct responses were “same” and the other half were “different”). When the correct answer was “different”, the color combinations had changed by one color (92% of changes) or two colors (8% of changes). Irrelevant colors blue and green were also equally and randomly distributed in both tasks. All participants successfully completed the CMT-balloon task prior to taking part in the fMRI study with the CMT-clown task.

A total of 24 task blocks (168 task trials) and 24 control blocks were presented. The task blocks were in four successive runs of six 32 sec blocks, each containing eight stimuli. Each block contained only one difficulty level; all difficulty levels were presented in pseudo-random order within each of the four runs. Total task time per difficulty level was 4 × 32 sec = 128 sec. The top of Figure 1 shows a sequence of task blocks alternating with control blocks. Participants had 3 sec to view a figure and respond, followed by 1 sec interstimulus interval during which a central plus sign (+) was presented.

Control blocks were 16 sec long each (Fig. 1). “Control 1” was a fixation cross; “control 2” was four different clown figures colored blue and green (3 sec each) interleaved by a plus sign (1 sec); and “control 3” was four clown figures as control 2, with a dot appearing at different locations within the clown figure every second to encourage attentional and/or eye movements. Control 2 and 3 were interleaved with plus signs to resemble the main task in visual–spatial features. Control blocks were presented after every task block in a pseudo-random order. Total block time per control type was 128 sec (2 × 4 × 16 = 128 sec), that is, equal to total task time per difficulty. Every run began and ended with a 10 sec presentation of the fixation cross.

Accuracy and response times were recorded; items were correct when responded to correctly within 3 sec. Working memory capacity score corresponded to the highest difficulty level reached with 70% accuracy (i.e., 20 of 28) or better, given similarly reliable performance on lower levels (Arsalidou et al. 2010 for details). A proportion correct score was calculated for the CMT that included only task blocks successfully completed with 70% or more correct (i.e., five or more items of seven for each block).

#### Figural intersections task

Figural intersections task (FIT) is a task with graded levels of difficulty, established to measure working memory capacity (mental/voluntary attention – Pascual-Leone and Baillargeon 1994), used here as additional behavioral task. It contains 2–8 geometric shapes presented separately on the right side of a page and overlapping on the left side. Participants were asked to attend to the shapes on the right and locate the shapes' total intersection available in the compound left-side figure. The number of relevant shapes in an item gives its task demand. There are seven levels of difficulty presented in 42 randomly ordered items (six items per level). Working memory capacity score corresponds to the highest difficulty level passed with at least 66% correct (i.e., 4 of 6). Behavioral responses to this task were used in correlations with performance on the CMT and fMRI signal change.

### Image acquisition

All images were acquired using an eight-channel head coil on 1.5T GE Excite HD scanner (GE Medical Systems, Milwaukee, WI). As anatomical reference, a set of high-resolution T1-weighted axial three-dimensional (3-D) SPGR images, covering the whole brain, were acquired first (116 slices; TR/TE/FA = 9 msec/4.2 msec/15°; voxel size = 0.9375 × 0.9375 × 1.5 mm, 2 NEX, 6 min). Then, functional images were acquired using a two-dimensional (2-D) spiral in–out imaging sequence as it provides better signal in the prefrontal regions (Preston et al. 2004); TR/TE/FA = 2 sec/40 msec/90°, voxel size = 3.75 × 3.75 ×5 mm) over 24 contiguous axial slices. Visual stimuli for the functional task were displayed centrally within the participant's visual field (12.4° horizontal, 16.5° vertical) on an MR-compatible goggle projection system (Resonance Technologies Inc., Los Angeles, CA). Participants responded to trials using an MR-compatible keypad (Lumitouch, Photon Control Inc., Burnaby, BC, Canada), pressing one key for “same” and another key for “different” with their right hand. Stimuli were controlled and responses recorded using the software Presentation (Neurobehavioural Systems Inc., Albany, CA).

### Analysis of behavioral data

Accuracy (proportion correct) and response times were calculated for each difficulty level; two repeated-measures ANOVAs were performed to examine differences among difficulty levels for accuracy and response times. To examine construct validity, we performed correlations among behavioral task scores (CMT-clown, CMT-balloon, and FIT) and correlations between brain activity and tasks administered outside the scanner (CMT-balloon and FIT). Importantly, because these analyses were testing construct validity, correlations were computed on average group scores across difficulty levels.

### fMRI analysis

Preprocessing and analyses of functional data were performed using AFNI (Cox 1996). Functional images were reconstructed into 3-D space and coregistered with the anatomical reference images. The first three volumes were discarded to allow for signal intensity equilibration. After motion correction (all participants moved <1 voxel), images were smoothed using a 3-D Gaussian filter (RMSD 8 mm). Images were spatially normalized to the MNI N27 brain in Talairach stereotaxic space and resampled to 3-mm cubic voxels. In lieu of high pass filtering, low-drift order was accounted by the model; the 3d-Deconvolve program in AFNI controls for temporal drift and autocorrelations. Data were fit to a block design general linear model using the task parameters of successful blocks (e.g., control blocks: c1, c2, and c3; and difficulty levels: D3, D4, D5, D6, D7, and D8) as variables of interest for each participant; failed blocks (accuracy <70%) were also accounted for by the model, but not used in the analyses. An accuracy of ≥70% was selected as criterion because it is also the percentage of accuracy per difficulty level used to calculate working memory capacity for each individual child (Arsalidou et al. 2010) and for adults. This criterion permits the elimination of instances of chance performance, which varies over difficulty levels, without having to exclude participants – which would affect statistical power. This method of substantiating task compliance allows for inclusion of trials with consistent task performance within a block. Following selection of attained blocks, a statistical parametric map was produced for each participant, indicating brain regions associated with each difficulty level and each control. Across all participants, there were 0, 2, 4, 3, 11, and 21 blocks failed for difficulty levels D3, D4, D5, D6, D7, and D8, respectively.

Individual results were then introduced into group analyses using random*-*effects analysis of variance. To examine the relation among difficulty levels, linear trend analyses were performed on task difficulty minus control (D-c) contrasts, for each control (e.g., D3-c2 < D4-c2 <D5-c2 < D6-c2 < D7-c2 < D8-c2). To correct for multiple comparisons, significant activations are reported using False Discovery Rate (FDR; Logan and Rowe 2004) at *q* < 0.05.

Simple contrasts conducted between difficulty levels and controls (e.g., D3-c1) were used to decompose the pattern of linearity in regions obtained from the linear trend analyses. Central regions of interest (ROIs) were selected from activations and deactivations obtained using the linear trend analyses. Average percent signal change and standard error scores were extracted from (ROIs; 6 mm in diameter, a total of eight voxels) and plotted against difficulty level. Of these ROIs, we illustrate a selected group of regions commonly classified into either working memory (e.g., Owen et al. 2005) or default-mode areas (Spreng et al. 2009). All ROIs, however, were used to compute correlations.

Behavioral scores (e.g., proportion correct and response times) were correlated with percent signal change in each ROI for each difficulty level (e.g., D3-c2, D4-c2, …, D8-c2). These correlations were performed with signal change and behavioral scores (obtained outside the scanner) averaged across participants for each difficulty level.

## Results

### Task performance

**Figure 2:**
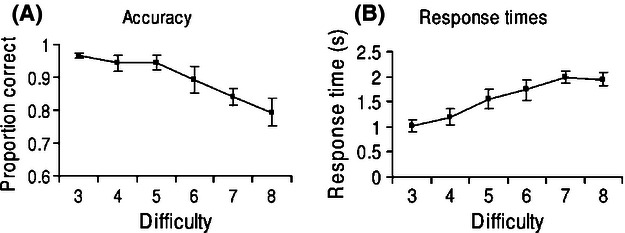
Behavioural performance on the color matching task (CMT)-clown. *X*-axis corresponds to difficulty level. (A) Mean proportion of correct for difficulty levels 3–8, passed with 70% or more correct responses, and standard error bars. (B) Mean response times for difficulty levels 3–8, passed with 70% or more correct responses.

Performance accuracy decreased as the number of colors to be remembered in the stimuli increased, the response time increasing concurrently (Fig. 2). There was a main effect of difficulty on accuracy (*F*_(5, 25)_ = 9.39, *MSE* = 0.003, *P* < 0.001, *partial η*^*2*^ = 0.65), but in post hoc tests, the only significant accuracy difference was between difficulty level seven (D7) and our easiest difficulty level (D3) (Table 1). Thus, comparisons of brain activity related to difficulty levels were made under comparable accuracy scores across most levels. There was also a main effect of response times (*F*_(5, 25)_ = 35.68, *MSE* = 0.026, *P* < 0.001, *partial η*^*2*^ = 0.88), which was driven by a significant effect between D4 and D5. Follow-up tests are presented in Table 1. Based on the highest difficulty level passed, our participants were estimated to have a working memory capacity of 6.63 ± 1.41, consistent with theoretical predictions of a magical number 7 ± 2 (Miller 1956; Pascual-Leone 1970).

**Table 1:** CMT-clown: differences across difficulty levels

	D3		D4		D5		D6		D7	
					
	*d*	MD	*d*	MD	*d*	MD	*d*	MD	*d*	MD
A. Accuracy (proportion correct)										
D4	0.56	0.02								
D5	0.55	0.02	−0.04	0.00						
D6	1.04	0.08	0.63	0.05	0.67	0.05				
D7	2.81	0.13*	1.73	0.11	1.82	0.11	0.64	0.05		
D8	2.26	0.18	1.74	0.15	1.78	0.15	0.94	0.10	0.52	0.05
B. Response times (in sec)										
D4	−0.50	−0.17								
D5	−1.27	−0.52*	−0.81	−0.36*						
D6	−1.69	−0.71*	−1.21	−0.54	−0.37	−0.19				
D7	−3.21	−0.96*	−2.36	−0.79*	−1.06	−0.44	−0.59	−0.25		
D8	−2.91	−0.91*	−2.14	−0.75*	−0.92	−0.39	−0.47	−0.20	0.14	0.04

Difficulty levels (D3–D8). (A) Follow-up tests from ANOVA using accuracy scores. (B) Follow-up tests from ANOVA using response times. *d* represents Cohen's d (effect size); MD, mean difference.

* Significant at *P* < 0.05 corrected for multiple comparisons using Bonferroni.

Correlations among behavioral task scores and percent signal change from a sample of ROIs are presented in Table 2. These correlations were computed on average scores between the mean scores across item difficulty levels in our behavioral tasks, and the mean scores of activity in the cortical ROIs. An extended correlation table including all ROIs can be found in Table S1.

**Table 2:** Correlations among brain responses and behavioral performance

					Working memory						Default-mode		
			
	Tasks				CG	INF	MIDF		FFG	PREC	MEDF	STG	PCC
									
	AC	RTB^1^	AB^1^	FIT	R-BA32	R-BA9	R-BA46	R-BA6	R-BA19	R-BA7	R-BA10	R-BA42	L-BA31
RTC	−0.85*	0.99**	−0.96*	−0.77									
AC	—	−0.93*	0.99**	0.93**									
RTB^1^		—	−0.97**	−0.94*	0.98**	0.96*	0.98**	0.97**	0.97**	0.91*	−0.85	−0.78	−0.87
AB^1^			—	0.85	−0.98**	−0.94*	−0.97**	−0.96**	−0.95	−0.86	0.72	0.69	0.76
FIT				—	−0.89*	−0.81	−0.85*	−0.81*	−0.65	−0.74	0.95**	0.77	0.99**

As the linear patterns were similar across controls, signal change of difficulty levels minus control 2 was used. Observations correspond to difficulty levels, not individuals.

AB, accuracy CMT-balloon; AC, accuracy CMT-clown; CG, cingulate gyrus; FFG, fusiform gyrus; FIT, figural intersections task, an alternative measure of voluntary/mental attention; INF, inferior frontal gyrus; L, left hemisphere; MEDF, medial frontal gyrus; MIDF, middle frontal gyrus; PCC, posterior cingulate; PREC, precuneus; R, right hemisphere; RTB, reaction times CMT-balloon; RTC, reaction times CMT-clown; STG, superior temporal gyrus. An extended table with all ROIs can be found in the Supporting Information.

* Correlation significant at *P* = 0.05;

** Correlation significant at *P* = 0.01; 2-tailed, *N* = 6 because of six levels of difficulty (D3–D8), except.

1 *N* = 5 for correlations with CMT-balloon (D3–D7). Accuracy corresponds to proportion correct.

### Neuroimaging results

**Figure 3:**
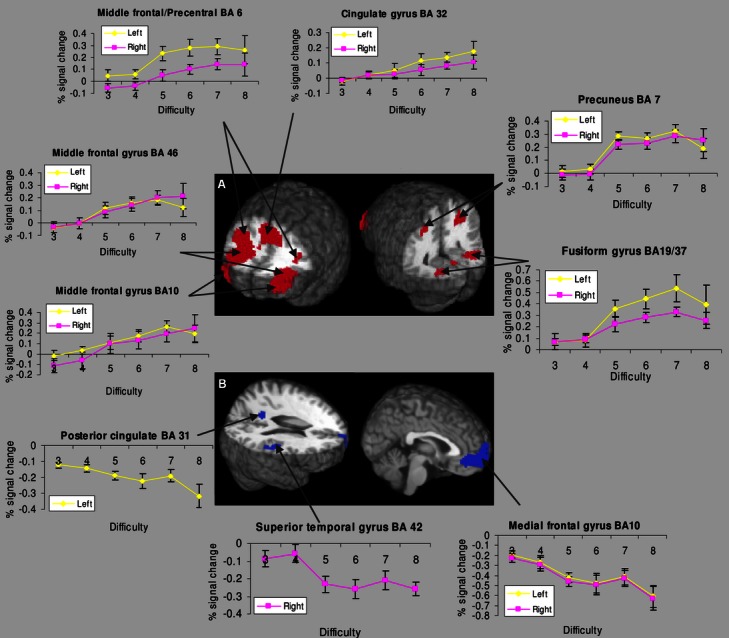
Brain areas that showed a linear trend as a function of difficulty. (A) Areas that increased in activity and (B) areas that decreased in activity. BA = Brodmann area. Significant activations are reported using False Discovery Rate at *q* < 0.05 for the linear contrast D3-c2 < D4-c2 < D5-c2 < D6-c2 < D7-c2 < D8-c2. Graphs represent percent signal change and standard error extracted from regions of interest (ROIs) (6 mm in diameter), centred over significantly active regions.

**Figure 4:**
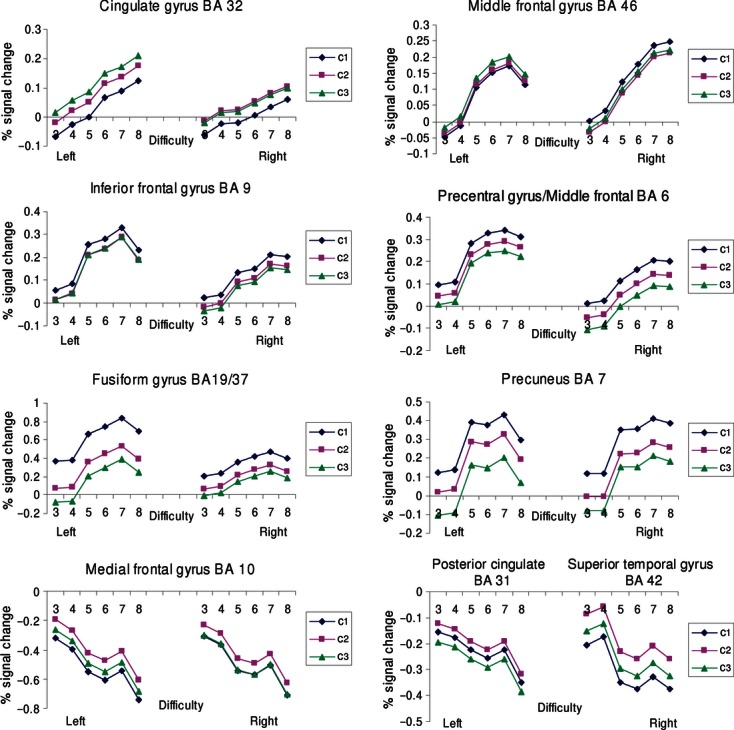
Changes in percent signal change as a function difficulty between task difficulty and control conditions.

Whole-brain activity was examined via linear trend analyses performed across comparisons of difficulty (D) levels (3–8) and one control – for each control condition (c: 1–3). The analyses tested these patterns: Trend 1 = D3-c1 < D4-c1 < D5-c1 < D6-c1 < D7-c1 < D8-c1; Trend 2 = D3-c2 < D4-c2 < D5-c2 < D6-c2 < D7-c2 < D8-c2; Trend 3 = D3-c3 < D4-c3 < D5-c3 < D6-c3 < D7-c3 <D8-c3. We report results for Trend 2 (Table 3; Fig. 3) because activated areas showing a linear relation with difficulty were very similar for the three trend analyses, as expected (Fig. 4; Table S2); small differences were attributable to different noise levels in the control conditions. These analyses showed that some brain areas increased in activity as a function of difficulty, while others decreased (Fig. 3). Even though, we did not anticipate a quadratic trend in the data, we tested this hypothesis and found no significant result.

**Table 3:** Linear changes in brain activity as a function of difficulty

volume (mL)	*x*	*y*	*z*	*t*-value	Hemisphere	Area	BA
Task activations: working memory							
94.26	14	26	26	3.76	R	Cingulate gyrus	32
X	−5	20	36		L	Cingulate gyrus	32
X	40	6	23		R	Inferior frontal gyrus	9
X	−39	8	28		L	Inferior frontal gyrus	9
X	39	30	31		R	Middle frontal gyrus	9
X	34	45	24		R	Middle frontal gyrus	10
X	−39	49	13		L	Middle frontal gyrus	10
X	42	28	22		R	Middle frontal gyrus	46
X	−40	39	18		L	Middle frontal gyrus	46
X	31	4	47		R	Middle frontal gyrus	6
X	−40	4	34		L	Precentral gyrus	6
X	32	20	1		R	Insula	13
X	−29	23	9		L	Insula	13
59.00	−3	−63	−11	3.57	L	Declive	
X	−36	−56	−13		L	Fusiform gyrus	37
X	25	−58	−9		R	Fusiform gyrus	39
14.20	21	−61	43	3.91	R	Precuneus	7
X	35	−48	41		R	Inferior parietal lobule	40
7.88	−22	−60	42	3.82	L	Precuneus	7
6.62	7	−21	6	3.30	R	Thalamus	
0.08	−8	−84	−20	3.23	L	Declive	
0.05	−3	−86	−16	3.07	L	Declive	
0.05	30	−83	33	3.22	R	Cuneus	19
Control activations: default mode							
2.40	−1	49	1	3.62	L	Medial frontal gyrus	10/32
X	−3	43	1		L	Anterior cingulate	32
X	4	50	1		R	Medial frontal gyrus	10
2.40	53	−23	17	3.22	R	Postcentral gyrus	40
X	59	−26	17		R	Superior temporal gyrus	42
0.38	−12	−52	24	3.11	L	Posterior cingulate	31
0.14	41	11	−20	3.04	R	Superior temporal gyrus	38

Results from a linear contrast D3-c2 < D4-c2 < D5-c2 < D6-c2 < D7-c2 < D8-c2. Talairach coordinates in neurological convention represent the center of the cluster; *t*-value represents the mean *t*-value over that cluster. X = area within cluster. Results are controlled for multiple comparisons with False Discovery Rate (FDR) *q* = 0.05; BA = Brodmann area. Areas associated with working memory increased as a function of difficulty and areas associated with default mode decreased as a function of difficulty.

#### Activation increases with difficulty

Graphs of percent signal change with standard error for ROIs of activated brain regions showed a stepwise linear pattern in most regions (Fig. 3). These patterns did not change with control condition (Fig. 4). Magnitude of the signal change increased markedly with difficulty, particularly between D5 and D7, in bilateral middle prefrontal cortex (BA 46), middle prefrontal cortex (BA 10), cingulate gyrus (BA 32), right middle frontal gyrus (BA 6), left precentral gyrus (BA 6), bilateral fusiform gyrus (BA 19/37), and precuneus (BA 7) (Fig. 3A). In the prefrontal regions, a linear increase in activation with task difficulty, greater in the left hemisphere was seen up to D7, congruent with participants' average behavioral working memory capacity score, which was found to be close to seven units. The dorsal subdivision of the cingulate gyrus increased its activation up to and including level D8 – the most demanding level. In contrast, the posterior regions activated the precuneus in particular, showed largely a step function with increased activation in difficulty levels D5 through D8, in contrast to levels D3 and D4.

#### Activation decreases with difficulty

Activation found in medial and temporal brain regions decreased as a function of difficulty level (Fig. 3B). This decreasing pattern was found in bilateral medial prefrontal cortex (BA 10), posterior cingulate (BA 31), and superior temporal cortex (BA 42). Gradual decreases in percent signal change was more closely followed in the medial prefrontal cortex, than posterior regions (Fig. 3B). This finding parallels the observation of the differential increases in anterior and posterior areas related with working memory (discussed above) as a function of difficulty.

## Discussion

We found mutually and inversely modulated linear relations between activity in areas associated with task difficulty (working memory or voluntary attention) and areas associated with control conditions (default mode) as a function of the difficulty levels in this task. The obtained functional relation between these activated regions suggests that these processes were not only complementary but also competitive or opposing each other in cognitively demanding situations.

### Working memory

We used a validated working memory capacity measure (Arsalidou et al. 2010) and found a group of brain areas to increase in activity as a function of difficulty. These regions were consistent with previous findings on working memory tasks (e.g., Rypma et al. 1999, 2002) and were in agreement with meta-analyses of working memory tasks (e.g., Owen et al. 2005), which show in adults that working memory is a multiregion process. We found activity to increase with difficulty in the prefrontal cortex, the cingulate gyrus as well as temporal and parietal regions. The prefrontal cortex plays a key role in working memory processes. Our data suggest that middle and inferior prefrontal gyri (implicated in executive function) may participate in keeping information “in mind” and analyzing relevant aspects of items, as these brain regions showed increasing activation with difficulty only up to the participants' average working memory capacity levels. This is in agreement with previous reports about hierarchical organization of prefrontal regions and their relations to executive functions (Christoff and Gabrieli 2000; Jonides et al. 2005). The cingulate gyrus also plays a key role in executive processes (Owen et al. 2005), with the dorsal subdivision associated with cognitive processes (Bush et al. 2000). The fact that activity in this region increased through to the highest level of difficulty supports the hypothesis that the cingulate gyrus is implicated in cognitive goal definition (Bush et al. 2000).

In addition to the cingulate gyrus, left and right insula areas were active and modulated by difficulty in our data; both regions have been associated with initiating motivated behaviors, as part of switching between working memory and default-mode processes (Sridharan et al. 2008; Uddin and Menon 2009). We also observed that activity in the right thalamus increased linearly as a function of difficulty. Thalamic gating affects cortico-thalamo-cortical (Nadeau 2008) and cortico-cortical communications (Sherman and Guillery 2002) and thus may help coordinate information coming from sensory as well as cortical sources. The fusiform gyri, which were active bilaterally, are associated with encoding object properties such as color, shape, and texture (e.g., Ungerleider and Mishkin 1982; Zeki and Marini 1998). The fusiform sources showed a marked increase between D4 and D5, with a more gradual increase to D7, suggesting considerably greater involvement in ventral stream processing when the task became more difficult. A similar step function between D4 and D5 was seen in precuneus activation, with a plateau between D5 and D8, which may suggest relatively constant recruitment (when difficulty is high) of this region that plays a key role in spatial attention and attention shifts (Nagahame et al. 1999; Vandenberghe et al. 2001).

Correlations among behavioral task scores, as well as correlations between behavioral scores collected outside the scanner and percent signal change were computed to appraise construct validity. As already suggested, these correlations were not computed across individuals; instead we correlated mean scores of behavioral working memory tests and mean activity in the cortical ROIs. We highlight the correlations between accuracy mean score of CMT-clown (AC score of Table 2) and FIT, vis-à-vis the obtained cortical activity in ROIs. Unlike the CMT, the FIT is a paper–pencil measure of working memory capacity across development (Pascual-Leone 1970; Pascual-Leone and Ijaz 1989; Pascual-Leone and Baillargeon 1994; Pascual-Leone et al. 2000). Scores from the FIT correlate highly with the ability to solve multiplication problems (Agostino et al. 2010) and was found to predict cognitive giftedness (Johnson et al. 2003; Pascual-Leone and Johnson 2005) and specific language impairment (Im-Bolter et al. 2006). The FIT predates the CMT and was chosen to evaluate its performance. In a developmental study, CMT and FIT were significantly correlated and yielded very similar quantitative working memory capacity scores (Arsalidou et al. 2010). In the current adult data, we also found that correlations between CMT-clown and FIT were very high (0.93) suggesting that these tasks are measures of the same latent variable. Response accuracy decreased with the cognitive demand (difficulty), even though the cortical activity in working memory regions increased with the items' cognitive load. Negative correlations (from −0.65 to −0.89) were obtained with percent signal change and the FIT, which was not studied with fMRI. This high negative relation using an alternative measure confirms that the pattern of cortical activity reflects a graded relation of covariation between activity in brain regions and the participants' use of working memory, which FIT has measured independently. An extended correlation table including all ROIs can be found in Supporting Information (Table S1).

Linear trend analyses showed that several regions congruent with working memory processes become progressively active as cognitive load increases. The linear patterns, however, did not show the same signature. Areas in the prefrontal cortex gradually increase until about D7 and leveled off or decreased at D8, whereas posterior regions, such as the precuneus and fusiform gyri, produced a distinct increase between D4 and D5 with a more steady increase to D7. The cingulate gyrus, on the other hand, appeared to produce its own pattern with activity progressing gradually up to the highest level of difficulty. We compare these patterns to those produced by areas that showed a decrement in activity as cognitive load increased, related to the default mode. Implications of this finding with reference to working memory capacity measurement are discussed in the section on capacity limits of working memory.

### Default mode

The coordinated deactivation in regions linked to the control task was also linear, supporting the hypothesis of an inverse regulation between default-mode and working memory processes (Raichle and Gusnard 2002), and this relation was maintained across increasing difficulty levels (McKiernan et al. 2003). Although our control tasks/baselines do not represent a pure resting state, they carried very limited cognitive demand, and responses induced by sensory processing disrupt only minimal activity in default-mode areas (Greicius et al. 2003). Our obtained linear patterns (Fig. 4) agree with these results.

Areas that decreased in activity as a function of difficulty were medial prefrontal cortex, posterior cingulate, and superior temporal gyri, which have been linked with self-relevant thoughts, integrating information, and memory associations, respectively (Buckner et al. 2008). Based on the need to suppress distracting cues and to process relevant colors as difficulty increased, more cognitive resources had to be dedicated to problem solving, rather than mind wandering. Behavioral performance from an independent working memory capacity measure, the FIT was significantly correlated with brain activity in these regions (Table 2; Table S1).

Pattern differences also appeared among regions that showed significant decreases in activity with increasing cognitive load, although the differences were less prominent than those observed in areas associated with working memory. Frontal regions (medial prefrontal cortex) and the posterior cingulate showed a steady deactivation with difficulty, whereas the temporal cortex showed a distinct deactivation between D4 and D5. Correlations between behavioral scores obtained outside of the scanner and fMRI signal change indicate that control-task processes represent an underlying variable inversely related to task processes, perhaps expressing exchange of resources between working memory (executive control) processes and default-mode (automatic, effortless control) processes. This is consistent with recent work on individual differences that suggests that participants with a higher capacity of working memory showed a higher tendency to mind wander during cognitive activities (Levinson et al. 2012). Similarly, cognitive activities that employ partial resources to engage working memory leave some resources available for mind wandering, which would engage the default-mode areas. Thus, our testing of limits in working memory capacity yields some clarity about dynamic interrelations, interchange, or balance between working memory and default mode.

### Capacity limits of working memory

The number of items adults can hold in mind is debated (Miller 1956; Pascual-Leone 1970; Cowan 2005; Halford et al. 2007). We suggest that normal adults have two capacity limits: an upper bound or *reserve* of up to 7 items (Miller 1956; Pascual-Leone 1970; Pascual-Leone and Johnson 2005, 2011) and a lower bound, or usual functional level, of 4 or 5 units (Cowan 2005; Pascual-Leone and Johnson 2011). As our protocol design encompassed both of these limits (i.e., difficulty levels 3–8) our data can also be used to determine if these limits were valid constructs.

Although the relation between activation and task demand could be generally described by linear models, there were a number of areas that showed more of a step function. For instance, brain activity in the precuneus showed a sharp increase between difficulty 4 and 5 whereas the middle frontal gyri (BA 46) showed a steady increase up to difficulty 7 (Fig. 3). These effects suggest nonlinearities between task demand and regional brain activity. As our imaging data were highly correlated with our behavioral data (including FIT task); the effects may be indexing mobilization of different aspects of working memory capacity. Steady increases in activation with task difficulty were seen up to D7 in prefrontal regions (Fig. 3A), consistent with the obtained average behavioral scores (in both FIT and fMRI task) of about seven items in our participants. Both our behavioral and fMRI data suggest that working memory capacity can reach about seven items in adults, in contrast to Cowan's model of working memory for which 4 is the upper limit of capacity (Cowan 2005). This latter view is not without support; however, D5 was the least demanding level of items that strongly activated areas of visual–spatial processing (see Fig. 3A); that is, four items might be adults' lower bound of working memory. Congruently, behavioral data showed a significant difference (with a large effect size) between reaction times for difficulty level 4 and 5 (Cohen's *d* = 0.81; Table 1). These findings offer a deeper understanding of limits of working memory capacity (i.e., 4 as lower bound vs. 7 as upper bound; Miller 1956; Pascual-Leone 1970; Cowan 2005; Halford et al. 2007; Pascual-Leone and Johnson 2011).

### Limitations

In this study, we used a novel developmentally validated task to assess the neural correlates of working memory capacity in adults. While the present study shows how such approach can inform our understanding of brain-behavior relations, several limitations have to be considered. The small number of participants poses a drawback and as we were mindful of this issue we present data corrected for multiple comparisons using FDR. Another consideration in data evaluation was our criterion for block inclusion in statistical analyses. It is typical in experiments to analyze only trials with correct responses. In our experiment, we used block analyses and only used blocks that 70% or more correct trials. We were interested in capturing activity related to the process of solving the task and not activity elicited by potentially performing correctly at chance level. The 70% criterion was theoretically chosen and behaviorally it was found to control for blocks that were above and beyond the working memory capacity level of the participant. This criterion allows for inclusion of trials with consistent performance within a block. Lastly, the caveats of ROI-based statistics have been previously presented (e.g., Vul et al. 2009) and challenged (Lieberman et al. 2009). Although we extracted percent signal change from significant areas following whole-brain analyses, we only present independent correlations of brain activity with behavioral scores obtained outside the scanner. We remain circumspect about these limitations. Nonetheless, the possibility that a linear relation exists between brain activity elicited by variable working memory demand levels and corresponding levels of resting state is a novel finding that warrants replication and further research with developmental samples.

## Conclusions

Our results confirm and expand previous observations suggesting a balancing of processing resources in the brain, which occurs via reallocation. We have found trade-offs in dynamic activation between brain areas related to the (executive-driven) task versus those elicited by the control condition. We demonstrated a direct linear relation between task performance and difficulty, together with an inverse relation between areas serving working memory versus the default-mode systems. Using the terminology of William James (1892), these are areas serving voluntary attention versus automatic/spontaneous attention. Such a balancing act of the brain expresses executive coordination of activation versus inhibition in the cortex (Edelman and Tononi 2000), a coordination that is likely to be automatic (Berthoz 2002). Behavioral work shows that working memory capacity undergoes gradual improvements with age (Pascual-Leone and Johnson 2005, 2011; Morra et al. 2008; Arsalidou et al. 2010). We have found that our protocol's parametric variation of cognitive task difficulty can capture graded variations in working memory and default-mode functions in adults, and as this task was designed and validated for children, it would be suitable for future investigations of young populations with lower working memory capacity limits.

## Supporting Information

**Table S1.** Correlations among brain responses and behavioural performance.

**Table S2.** Linear changes in brain activity as a function of difficulty.
