# Supplementary material for: A balancing act of the brain: activations and deactivations driven by cognitive load
Source: Brain Behav. 2013 Apr 2;3(3):273–85. doi: 10.1002/brb3.128 (PMC3683287; doi:10.1002/brb3.128)
Supplement: Supplementary file 1 [file brb30003-0273-SD1.docx]

**Appendix**

**Table S1**

Correlations among brain responses and behavioural performance

| **A** | **Working memory: Frontal regions** | | | | | | | | | | | | | | | | | | | | | | | | | |
| --- | --- | --- | --- | --- | --- | --- | --- | --- | --- | --- | --- | --- | --- | --- | --- | --- | --- | --- | --- | --- | --- | --- | --- | --- | --- | --- |
|  | CG | | | | | INF | | | | | MIDF | | | | | | | | | | | | | | PRC | |
|  | L-BA32 | | R-BA32 | | | L-BA9 | | R-BA9 | | | R-BA9 | | L-BA10 | | | R-BA10 | | | L-BA46 | R-BA46 | | | R-BA6 | | L-BA6 | |
| RTB**†** | 0.99** | | 0.98** | | | 0.95* | | 0.96* | | | 0.99** | | 0.97** | | | 0.98** | | | 0.97* | 0.98** | | | 0.97** | | 0.93* | |
| AB**†** | -0.98** | | -0.98** | | | -0.90* | | -0.94* | | | -0.97** | | -0.91* | | | -0.99** | | | -0.92* | -0.97** | | | -0.96** | | -0.88 | |
| FIT | -0.89* | | -0.89* | | | -0.57 | | -0.81 | | | -0.87* | | -0.88* | | | -0.69 | | | -0.63 | -0.85* | | | -0.81* | | -0.71 | |
| **B** | **Working memory: Posterior regions** | | | | | | | | | | | | | | | | | | | | | | | | |  |
|  | FFG | | | | | | PREC | | | | | IPL | | | Insula | | | | | | Declive | | | THA | |  |
|  | L-BA37 | | | R-BA19 | | | L-BA7 | | R-BA7 | | | R-BA40 | | | L-BA13 | | R-BA13 | | | | L | | | R | |  |
| RTB**†** | 0.95* | | | 0.97** | | | 0.90* | | 0.91* | | | 0.96* | | | 0.85 | | 0.94* | | | | 0.96* | | | 0.96** | |  |
| AB**†** | -0.92* | | | -0.95 | | | -0.83 | | -0.86 | | | -0.93* | | | -0.82 | | -0.93* | | | | -0.97** | | | -0.99** | |  |
| FIT | -0.63 | | | -0.65 | | | -0.48 | | -0.74 | | | -0.78 | | | -0.44 | | -0.78 | | | | -0.37 | | | -0.56 | |  |
| **C** | | **Default-mode: All regions** | | | | | | | | | | | | | | | | | | | | | | | |  |
|  | | MEDF | | | | | | | | ACC | | | | POSTC | | | | STG | | | | PCC | | | |  |
|  | | L-BA10 | | | R-BA10 | | | | | L-BA32 | | | | R-BA40 | | | | R-BA42 | | | | L-BA31 | | | |  |
| RTB**†** | | -0.89* | | | -0.85 | | | | | -0.88* | | | | -0.73 | | | | -0.78 | | | | -0.87 | | | |  |
| AB**†** | | 0.77 | | | 0.72 | | | | | 0.76 | | | | 0.63 | | | | 0.69 | | | | 0.76 | | | |  |
| FIT | | 0.95** | | | 0.95** | | | | | 0.96** | | | | 0.75 | | | | 0.77 | | | | 0.99** | | | |  |

Notes: A test of construct validity would examine whether signal change from areas underlying working memory and default-mode correlate with other working memory-capacity measures, such as FIT, and with performance (proportion correct and response times) in the CMT –Balloon, administered outside the MR scanner, across the six levels of difficulty. Since the linear patterns were similar across controls, signal change of difficulty levels minus control 2 was used. Observations here correspond to difficulty levels, not individuals. * Correlation significant at *p* = 0.05; ** Correlation significant at *p* = 0.01; 2-tailed, N= 6 because of six levels of difficulty (D3- D8), except †N=5 for correlations with CMT-Balloon (D3-D7). Accuracy corresponds to proportion correct. AB– accuracy CMT-Balloon; ACC– anterior cingulate; CG– cingulate gyrus; FFG– fusiform gyrus; FIT – Figural intersections task, an alternative measure of working memory capacity; INF– inferior frontal gyrus; IPL – inferior parietal lobule; L– left hemisphere; MEDF– medial frontal gyrus; MIDF– middle frontal gyrus; PCC– posterior cingulate; POSTC– postcentral gyrus; PRC – precentral gyrus; PREC– precuneus; R– right hemisphere; RTB– reaction times CMT-Balloon; STG– superior temporal gyrus; THA– thalamus

**Table S2**

Linear changes in brain activity as a function of difficulty

| A. Linear contrast: D3-c1 < D4-c1 < D5-c1 < D6-c1 < D7-c1 < D8-c1 | | | | | | | |
| --- | --- | --- | --- | --- | --- | --- | --- |
| **Working memory** | | | | | | | |
| volume (ml) | x | y | z | t-value | Hem. | Area | BA |
| 8.67 | 31 | 27 | 25 | 4.19 | R | Middle frontal gyrus | 9 |
| X | 40 | 33 | 15 |  | R | Middle frontal gyrus | 46 |
| 7.99 | 32 | 51 | 13 |  | R | Middle frontal gyrus | 10 |
| X | -6 | 19 | 40 |  | L | Cingulate gyrus | 32 |
| 1.70 | 3 | 22 | 35 |  | R | Cingulate gyrus | 32 |
| 1.54 | 28 | 7 | 45 |  | R | Middle frontal gyrus | 6 |
| 1.51 | 34 | 22 | 3 |  | R | Insula | 13 |
| 1.08 | 25 | -59 | 40 | 3.98 | R | Precuneus | 7 |
| 1.03 | 39 | -53 | 40 |  | R | Inferior parietal lobule | 40 |
| 0.81 | -37 | 48 | 16 | 3.81 | L | Middle frontal gyrus | 10 |
| 0.35 | -41 | 38 | 21 |  | L | Middle frontal gyrus | 46 |
| 0.11 | -28 | 21 | 6 | 3.96 | L | Insula | 13 |
| 0.08 | 5 | -22 | 3 | 3.62 | R | Thalamus |  |
| 0.08 | -24 | -61 | 44 | 3.78 | L | Superior parietal lobule | 7 |
| 0.05 | 48 | -45 | -11 | 3.74 | R | Fusiform gyrus | 37 |
| 8.67 | -20 | -60 | -29 | 3.58 | L | Pyramis |  |
| X | -38 | 2 | 36 | 3.59 | L | Precentral gyrus | 6 |
| 7.99 | -18 | 4 | 46 | 3.49 | L | Cingulate gyrus | 24 |
| X | -9 | -69 | 45 | 3.52 | L | Precuneus | 7 |
| 1.70 | -28 | -30 | -22 | 3.63 | L | Culmen |  |
| 1.54 | 39 | -54 | -13 | 3.46 | R | Fusiform Gyrus | 37 |
| 1.51 | -24 | -29 | -28 | 3.43 | L | Culmen |  |
| **Default-mode** | | | | | | | |
| 1.89 | -1 | 50 | 0 | 3.65 | L | Medial frontal gyrus | 10 |
| X | 3 | 51 | -2 |  | R | Anterior cingulate | 10 |
| 0.08 | 48 | -13 | 15 | 3.44 | R | Insula | 13 |

| B. Linear contrast: D3-c3 < D4-c3 < D5-c3 < D6-c3 < D7-c3 < D8-c3 | | | | | | |  |
| --- | --- | --- | --- | --- | --- | --- | --- |
| **Working memory** | | | | | | | |
| volume (ml) | x | y | z | t-value | Hem. | Area | BA |
| 106.03 | 13 | 27 | 25 | 3.79 | R | Anterior cingulate | 32 |
| X | -6 | 19 | 40 |  | L | Cingulate gyrus | 32 |
| X | -29 | -2 | 46 |  | L | Middle frontal gyrus | 6 |
| X | 28 | -3 | 54 |  | R | Middle frontal gyrus | 6 |
| X | -40 | 33 | 24 |  | L | Middle frontal gyrus | 46 |
| X | 47 | 32 | 23 |  | R | Middle frontal gyrus | 46 |
| X | -35 | 50 | 10 |  | L | Middle frontal gyrus | 10 |
| X | 40 | 49 | 18 |  | R | Middle frontal gyrus | 10 |
| X | -41 | 5 | 28 |  | L | Inferior frontal gyrus | 9 |
| X | 46 | 10 | 22 |  | R | Inferior frontal gyrus | 9 |
| X | -29 | 21 | 8 |  | L | Insula | 13 |
| X | 32 | 22 | 3 |  | R | Insula | 13 |
| 56.78 | -1 | -64 | -12 | 3.58 | L | Declive |  |
| X | -41 | -65 | -12 |  | L | Fusiform gyrus | 19/37 |
| X | 35 | -53 | -12 |  | R | Fusiform gyrus | 37 |
| X | 28 | -81 | 11 |  | R | Middle occipital gyrus | 19 |
| 15.53 | 25 | -60 | 42 | 3.59 | R | Precuneus | 7 |
| X | 37 | -48 | 41 |  | R | Inferior parietal lobule | 40 |
| 8.10 | -24 | -60 | 42 | 3.74 | L | Precuneus | 7 |
| 5.75 | 6 | -22 | 5 | 3.24 | R | Thalamus |  |
| 0.14 | 32 | -42 | -34 | 3.11 | R | Cerebellar tonsil |  |
| 0.11 | 4 | -77 | -34 | 3.51 | R | Uvula |  |
| 0.08 | -26 | -79 | 7 | 3.00 | L | Middle occipital gyrus | 18 |
| 0.05 | -6 | -14 | 18 | 3.03 | L | Thalamus |  |
| 0.05 | 38 | -74 | 37 | 3.21 | R | Precuneus | 7 |
| 0.05 | 47 | -53 | 49 | 3.06 | R | Inferior parietal lobule | 40 |
| **Default-mode** | | | | | | | |
| 6.59 | -2 | 48 | 0 | 3.56 | L | Anterior cingulate | 32 |
| X | -1 | 59 | 5 |  | L | Medial frontal gyrus | 10 |
| X | 4 | 50 | 0 |  | R | Medial frontal gyrus | 10 |
| 2.05 | 45 | -19 | 17 | 3.17 | R | Insula | 13 |
| X | 47 | -28 | 17 |  | R | Superior temporal gyrus | 41 |
| 0.43 | -51 | -63 | 23 | 3.11 | L | Middle temporal gyrus | 39 |
| 0.05 | -39 | 14 | -19 | 3.06 | L | Superior temporal gyrus | 38 |

Notes: Talairach coordinates in neurological convention represent the center of the cluster; t-value represents the mean t-value over that cluster. X = area within cluster. Results are controlled for multiple comparisons with FDR *q* = 0.05; BA = Brodmann Area. Areas associated with working memory increased as a function of difficulty and areas associated with default-mode decreased as a function of difficulty.
